# Static and dynamic light scattering by red blood cells: A numerical study

**DOI:** 10.1371/journal.pone.0176799

**Published:** 2017-05-04

**Authors:** Johannes Mauer, Matti Peltomäki, Simón Poblete, Gerhard Gompper, Dmitry A. Fedosov

**Affiliations:** Theoretical Soft Matter and Biophysics, Institute of Complex Systems and Institute for Advanced Simulation, Forschungszentrum Jülich, 52425 Jülich, Germany; University of California Santa Barbara, UNITED STATES

## Abstract

Light scattering is a well-established experimental technique, which gains more and more popularity in the biological field because it offers the means for non-invasive imaging and detection. However, the interpretation of light-scattering signals remains challenging due to the complexity of most biological systems. Here, we investigate static and dynamic scattering properties of red blood cells (RBCs) using two mesoscopic hydrodynamics simulation methods—multi-particle collision dynamics and dissipative particle dynamics. Light scattering is studied for various membrane shear elasticities, bending rigidities, and RBC shapes (e.g., biconcave and stomatocyte). Simulation results from the two simulation methods show good agreement, and demonstrate that the static light scattering of a diffusing RBC is not very sensitive to the changes in membrane properties and moderate alterations in cell shapes. We also compute dynamic light scattering of a diffusing RBC, from which dynamic properties of RBCs such as diffusion coefficients can be accessed. In contrast to static light scattering, the dynamic measurements can be employed to differentiate between the biconcave and stomatocytic RBC shapes and generally allow the differentiation based on the membrane properties. Our simulation results can be used for better understanding of light scattering by RBCs and the development of new non-invasive methods for blood-flow monitoring.

## Introduction

Light scattering is commonly used in the fields of condensed, soft, and biological matter to investigate the structure and dynamics of different constituents within a material sample [1–3]. Examples include micro- and nano-particle suspensions [4–7], suspensions of fd virus [8], red blood cells (RBCs) [9–11], and skeletal-muscle contraction [12]. In the biomedical field, light scattering offers promising prospects of non-invasive imaging and monitoring of certain medical conditions without the necessity of contrast agents or radiation doses [13]. However, the interpretation of scattering signals is often cumbersome due to a complex nature of biological systems and standard theoretical models for light scattering used in colloidal science fail to provide reliable information. This motivates the development of realistic simulation models to deliver tools for an adequate interpretation of light-scattering measurements.

An important example of biological fluids attractive for the application of light scattering is blood. The major cell component of blood is RBCs, which constitute about 45% of blood by volume. RBCs contain a dense hemoglobin solution, which is believed to be the primary component for the scattering and absorption of the UV, blue, and green spectral ranges of light [14]. Different light scattering techniques have been already used to measure various properties of single RBCs [10, 11, 15, 16]. For example, static light scattering (SLS) has been employed to measure shape changes of RBCs in malaria [10] and sickle-cell [16] diseases. Furthermore, dynamic light scattering (DLS) has been employed to monitor RBC membrane fluctuations [10, 11], whose strength and dynamics can be related to cell’s mechanical properties and metabolic activity [17, 18]. Most of these measurements have been performed for RBCs lying on a coverslip such that the cell’s orientation remains fixed for the duration of experiments. However, there is a growing interest in light scattering measurements of RBCs in a suspension with and without flow [19, 20], but their quantitative interpretation remains challenging.

Numerical simulations have the potential to fill this gap and provide reliable tools for the quantitative interpretation of light-scattering measurements. Some examples include simulations of light scattering by single [21–23] and multiple [24, 25] RBCs and by cell organelles [26, 27]. All these studies have focused on SLS, which mainly allows the characterization of cell and organelle shapes. In this work, we make a step further and investigate SLS and DLS of a single diffusing RBC in order to access dynamical properties of these cells. This setup mimics light-scattering experiments performed on a dilute solution of RBCs.

RBC modeling has gained popularity in the last decade [28, 29], and several existing RBC models [30–32] are already able to deliver quantitative results in agreement with available experimental measurements. We model a RBC freely diffusing in a solvent and compute its average SLS and DLS properties. We employ the Rayleigh-Gans-Debye approximation [1] for the calculation of scattering amplitude, which is considered to be appropriate for small ratios of the refractive indices between a scatterer (here a RBC) and the surrounding medium. The static scattering function, averaged over many possible orientations of a diffusing RBC, is shown to be not very sensitive to the changes in RBC membrane properties, such as shear elasticity and bending rigidity, and to moderate changes in RBC shapes (e.g., biconcave and stomatocyte). Thus, the orientationally-averaged SLS measurements are expected to be impractical. However, the diffusive behavior of a RBC and its membrane properties can be detected by DLS measurements represented by the temporal correlations of instantaneous scattering amplitudes. We investigate DLS for RBCs with different membrane properties and cell shapes, and for various membrane-model discretizations. We find that the changes in RBC shape are detectable by the DLS measurements. The changes in membrane properties appear to hardly affect the diffusive behavior of the whole cell, as long as the cell shape remains nearly unaffected. Nevertheless, the DLS results for various membrane parameters show significant differences at high enough frequencies. This indicates that such measurements can be used to identify the membrane properties.

The paper is organized as follows. In Sec. Theoretical Background, we describe a theoretical background for the calculation of scattering amplitude and SLS and DLS functions. Section Methods and Models presents two mesoscopic hydrodynamics approaches, multi-particle collision dynamics [33, 34] and dissipative particle dynamics [35, 36], which we use in simulations. In the same section, we present a RBC model and simulation setup and conditions. Section Results contains the main results including SLS and DLS by a diffusing RBC for different membrane properties. In Sec. Discussion, we discuss the importance and limitations of our simulation results and conclude. Finally, appendices provide all necessary technical details for the calculation of scattering amplitudes over triangulated surfaces and for orientational averaging.

## Theoretical background

For the calculation of light scattering by RBCs, the Rayleigh-Gans-Debye approximation [1] is employed, which assumes elastic scattering such that the wave length is not altered by the scattering process. The incident electric-field amplitude of the light source and the scattering strength of a scatterer are set to unity considering that no light absorption occurs. The wave lengths inside and outside the scatterer are assumed to be equal and thus, no phase shift is present due to different path lengths through the scatterer. Phase shifts and interference can occur only due to different positions of the scattering objects. In a further approximation, the incident light is considered as a plane wave having a unique direction. This condition excludes refraction on the surface of scattering objects.

The instantaneous scattering amplitude *A*(**q**, *t*) by a solid object enclosing a volume *V*(*t*) at time *t* is then given by

$$\begin{array}{c} A\left(q,t\right)=\int_{V(t)}e^{iq\cdot x}{\text{d}}^{3}x\;, \end{array}$$ (1)

where **q** is the vector of momentum transfer. From the amplitude, the intermediate scattering function (ISF) *I*(**q**, *t*) is commonly measured in order to identify particle dynamics. It is defined as a correlation function of scattering amplitudes with a lag time *t*,

$$\begin{array}{c} I\left(q,t\right)={\left\langle A\left(q,t_{0}\right)A^{*}\left(q,t_{0}+t\right)\right\rangle }_{t_{0}}\;, \end{array}$$ (2)

where A*() denotes a complex conjugate and 〈〉_*t*_0__ is the time average. This function provides information about the dynamics of the object at different length scales. From *I*(**q**, *t*) at *t* = 0, we obtain the SLS function *I*(**q**, 0), which contains information about the shape of the object. Furthermore, the ISF *I*(**q**, *t*) can be used to derive the translational diffusion coefficient *D*_*T*_ of the object as

$$\begin{array}{c} D_{T}=\lim_{q\to 0}\lim_{t\to 0}\frac{1}{q^{2}}\frac{\partial \ln I(q,t)}{\partial t}\;. \end{array}$$ (3)

This relation can be generalized to obtain an effective diffusion coefficient *D*_eff_(*q*) defined as the *q*-dependent function

$$\begin{array}{c} D_{\text{eff}}\left(q\right)=\lim_{t\to 0}\frac{1}{q^{2}}\frac{\partial \ln I(q,t)}{\partial t}\;. \end{array}$$ (4)

The function *D*_eff_(*q*) characterizes diffusional properties and shape fluctuations of the object at non-zero *q* values.

In experiments, the time-dependent scattering intensity *I*_*int*_(**q**, *t*) = *A*(**q**, *t*)A*(**q**, *t*) is usually measured, from which the intensity correlation function $g_{I}\left(q,t\right)={\langle I_{int}\left(q,t_{0}\right)I_{int}^{*}\left(q,t_{0}+t\right)\rangle }_{t_{0}}$ is obtained. The correspondence between *g*_*I*_(**q**, *t*) and *I*(**q**, *t*) is provided by the Siegert relation as [1]

$$\begin{array}{c} g_{I}\left(q,t\right)=I{\left(q,0\right)}^{2}+{\left|I\left(q,t\right)\right|}^{2}. \end{array}$$ (5)

Further, we will present results in terms of the ISF, from which the intensity correlation function can easily be obtained.

**Fig 1:**
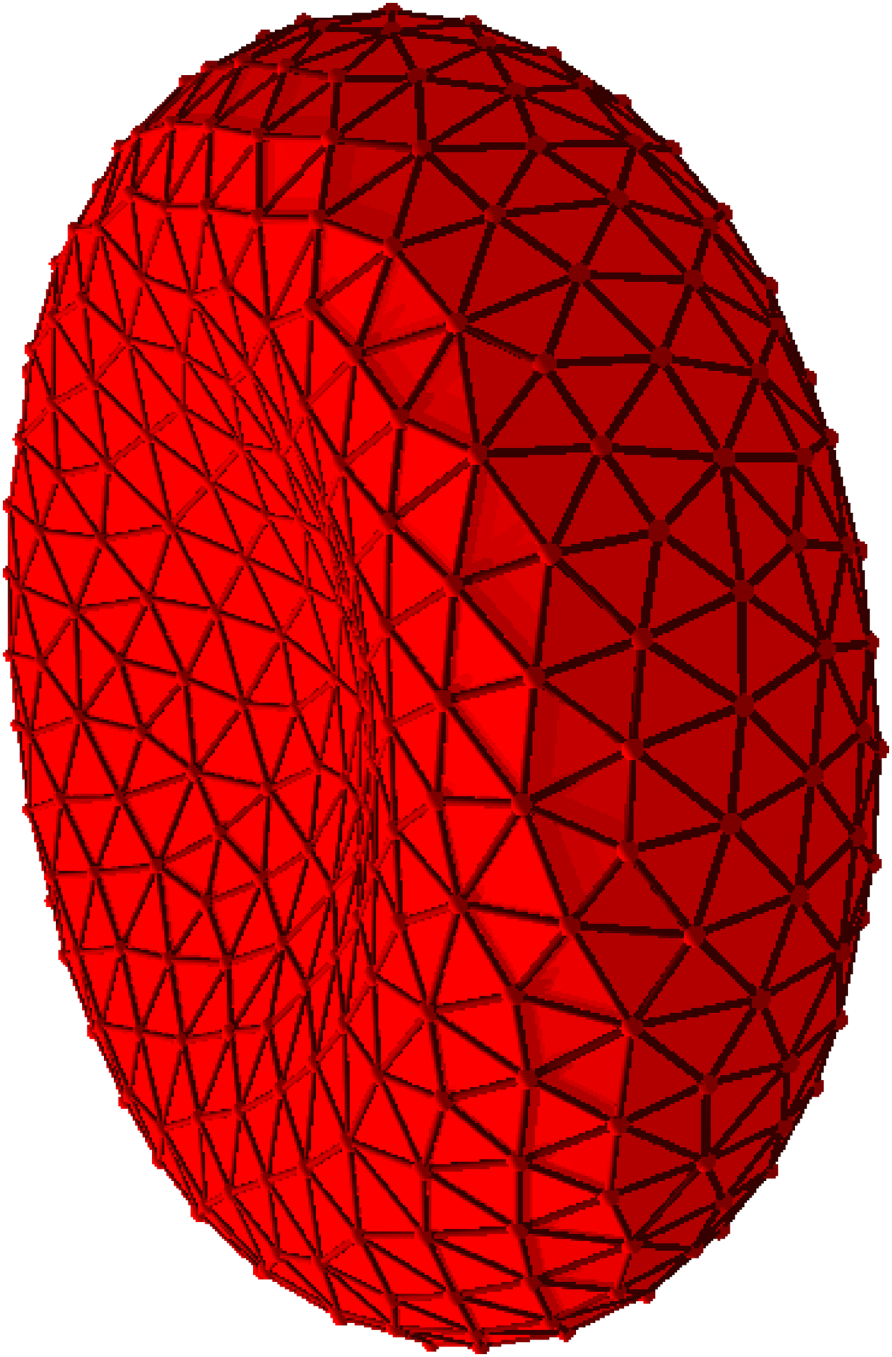
RBC membrane model. Conformation of a RBC membrane modeled as a closed triangulated surface. *N*_*m*_ = 500.

In simulations, it is more convenient to evaluate surface integrals instead of volume integrals, since RBCs and other shapes are modeled as triangulated closed surfaces, see Fig 1 and Sec. Methods and models for details. Thus, the instantaneous scattering amplitude in Eq (1) is calculated through an integral over the RBC surface, as described in S1 Appendix. It is also important to realize that the evaluation of the ISF in Eq (2) depends on the three-dimensional wave vector **q**. In order to characterize rotational diffusion properties of a RBC, measurements need to be performed over a time scale longer than the longest rotational-diffusion time of a RBC. Also, in a typical experimental setup, measurements would be performed simultaneously on multiple cells with different orientations (again due to the rotational diffusion). Therefore, the orientation of the cell with respect to the direction of the momentum change **q** becomes irrelevant. For this reason, we perform the orientational average of *I*(**q**, *t*) as

$$\begin{array}{c} I\left(q,t\right)=\frac{1}{4\pi q^{2}}\int_{|q|=q}I\left(q,t\right)\text{d}\Omega , \end{array}$$ (6)

which mimics different initial orientations of RBCs. In simulations, this integral is approximated by averaging *I*(**q**, *t*) over a large enough number of random vector orientations. Detailed description of this procedure is given in S2 Appendix.

## Methods and models

### Multi-particle collision dynamics

Multi-particle collision dynamics (MPC) [33, 34] is a mesoscopic hydrodynamics method, where a fluid is represented by a collection of *N*_*s*_ point particles with mass *m*_*s*_. Particle motion is advanced through two alternating steps: streaming and collision. In the streaming step, the MPC particles move ballistically without any interactions, i.e., their positions are updated according to

$$\begin{array}{c} r_{i}\left(t+\Delta t\right)=r_{i}\left(t\right)+\Delta tv_{i}\left(t\right)\;, \end{array}$$ (7)

where **r**_*i*_(*t*) and **v**_*i*_(*t*) are the position and velocity of a fluid particle *i* at time *t*, and Δ*t* is the collision time. In the collision step, the particles are binned into cells of a cubic lattice with a lattice constant *a*. Inside each cell *j*, all particles are subject to an instantaneous collision given by

$$\begin{array}{c} v_{i}^{\text{(new)}}\left(t\right)=v_{j,cm}+R\left({\hat{n}}_{j},\beta \right)\left(v_{i}\left(t\right)-v_{j,cm}\right)\;, \end{array}$$ (8)

where **v**_*j*,*cm*_ is the center-of-mass velocity of all particles in cell *j* and $R({\hat{n}}_{j},\beta )$ is an operator that rotates the relative velocities by an angle *β* around an axis given by the unit vector ${\hat{n}}_{j}$. Direction of the unit vector is chosen randomly and independently for all cells *j* and collision steps. Hence, this version of MPC is often called stochastic rotation dynamics (SRD). To keep Galilean invariance of a simulation system regardless of the choice for collision-cell lattice, a random grid shift is employed for every axis before each collision step [37].

A MPC solvent models a Newtonian fluid with a dynamic viscosity, which has two contributions: kinetic *η*_kin_ and collisional *η*_col_ [38, 39]. These two contributions can be computed as

$$\begin{array}{ccc} \eta_{\text{kin}} & = & \frac{nk_{B}T\Delta t}{a^{3}}\{\frac{n/B_{\beta }}{n-1+e^{-n}}-\frac{1}{2}\},\\ \eta_{\text{col}} & = & \frac{A_{\beta }m_{s}\left(n-1+e^{-n}\right)}{12a\Delta t}, \end{array}$$ (9)

where *k*_*B*_ *T* is the thermal energy with temperature *T*, *n* is the average number of solvent particles per collision cell, $A_{\beta }=\frac{2}{3}\left(1-\cos\left(\beta \right)\right)$, and *B*_*β*_ = 1 − cos (2*β*). The cell-level canonical sampling thermostat [40] has been employed in simulations, even though it is not necessary for equilibrium simulations.

### Dissipative particle dynamics

Another mesoscale hydrodynamics approach is the dissipative particle dynamics (DPD) method [35, 36], which is also a particle-based simulation technique. The main difference between DPD and MPC is the nature of particle interactions. In contrast to collisions in MPC, DPD particles interact through pairwise conservative, dissipative, and random forces given by

$$\begin{array}{ccc} F_{ij}^{C} & = & a_{c}w_{c}(r)e_{ij},\\ F_{ij}^{D} & = & -\gamma w^{2}(r)(e_{ij}\cdot v_{ij})e_{ij},\\ F_{ij}^{R} & = & \sigma w(r)\xi {\left(\Delta t\right)}^{-\frac{1}{2}}e_{ij}, \end{array}$$ (10)

where *a*_*c*_, *γ*, and *σ* are the conservative, dissipative, and random force coefficients, respectively. *w*_*c*_(*r*) and *w*(*r*) are distance-dependent weight functions defined as *w*_*c*_(*r*) = (1 − *r*/*r*_*c*_) and $w\left(r\right)=w_{c}^{k}\left(r\right)$, where *r* = |**r**|, *k* is a selected exponent, and *r*_*c*_ is the cutoff radius beyond which all interactions vanish. Furthermore, **e**_*ij*_ = **r**_*ij*_/*r*_*ij*_, *ξ* is a Gaussian random number with zero mean and unit variance, and Δ*t* is the time step.

The dissipative and random forces form a thermostat, which satisfies the fluctuation-dissipation theorem under the condition [36]

$$\begin{array}{c} \sigma^{2}=2\gamma k_{B}T. \end{array}$$ (11)

Time evolution of a DPD system follows the Newton’s second law which is integrated using the velocity-Verlet algorithm [41].

### Red blood cell model

The RBC membrane is modeled as a triangulated surface [30, 31, 42] with *N*_m_ membrane particles of mass *m*_m_. The number of bonds or edges *N*_*s*_ corresponds to 3*N*_m_ − 6, while the number of triangles or faces is equal to 2*N*_m_ − 4. Shear elasticity of the RBC membrane arises from the bonds connecting membrane particles with a harmonic spring potential

$$\begin{array}{c} U_{\text{spring}}=\sum_{ij\in E}\frac{k_{s}}{2}{\left(r_{ij}-r_{ij,0}\right)}^{2}, \end{array}$$ (12)

where *E* is the set of all springs in the triangulation, *r*_*ij*_ is the distance between membrane particles *i* and *j*, *r*_*ij*,0_ is the rest length of the spring *ij*, and *k*_*s*_ is the spring constant, which is directly related to the shear modulus *μ* of the membrane as $\mu =\sqrt{3}k_{s}/4$.

The curvature elasticity of a membrane is described by the bending energy

$$\begin{array}{c} U_{\text{bend}}=\frac{\kappa }{2}\int_{A_{0}}{\left(H_{1}+H_{2}-2C_{0}\right)}^{2}\;\text{d}A, \end{array}$$ (13)

where *κ* is the bending rigidity, *A*_0_ is the membrane surface area, *H*_1_ and *H*_2_ are the local principal curvatures, and *C*_0_ is the spontaneous curvature. The bending energy in Eq (13) with *C*_0_ = 0 can be discretized as [43, 44]

$$\begin{array}{c} U_{\text{bend}}=\frac{\kappa }{2}\sum_{i}\frac{1}{\sigma_{i}}{\left\{\sum_{j\in N(i)}\frac{\sigma_{ij}r_{ij}}{r_{ij}}\right\}}^{2}, \end{array}$$ (14)

where *N*(*i*) is the set of neighbors *j* of a membrane particle *i* in the triangulation, **r**_*ij*_ is the bond vector between vertices *i* and *j*, *r*_*ij*_ = |**r**_*ij*_|, *σ*_*ij*_ = *r*_*ij*_[cot(*θ*_1_) + cot (*θ*_2_)]/2 is the length of the bond between *i* and *j* in the dual lattice, *θ*_1_ and *θ*_2_ are the two angles opposite to the bond between *i* and *j* in the two triangles adjacent to that bond, and *σ*_*i*_ = ∑_*j*∈*N*(*i*)_ *σ*_*ij*_ *r*_*ij*_/4 is the area corresponding to membrane particle *i* in the dual lattice. The discretization in Eq (14) has been employed in MPC simulations, while in DPD simulations the simpler discretization was employed

$$\begin{array}{c} U_{\text{bend}}=\sum_{i\in 1...N_{s}}k_{b}[1-\cos(\theta_{i}-\theta_{0})], \end{array}$$ (15)

where *k*_*b*_ is the bending coefficient, *θ*_*i*_ is the instantaneous angle between two adjacent triangles having the common edge *i*, and *θ*_0_ is the spontaneous angle which can represent a non-zero spontaneous curvature of a membrane. The bending coefficient *k*_*b*_ can be expressed in terms of *κ* as $k_{b}=2\kappa /\sqrt{3}$. Generally, the discretization in Eq (14) is more accurate than that in Eq (15) [44]; however, it is more expensive computationally.

The area and volume of a RBC are constrained by the potentials

$$\begin{array}{ccc} U_{\text{area}} & = & \sum_{i\in F}\frac{1}{2}k_{\text{A}}{\left(A_{i}-A_{i,0}\right)}^{2},\\ U_{\text{vol}} & = & \frac{1}{2}k_{\text{V}}{\left(V-V_{0}\right)}^{2}, \end{array}$$ (16)

where *F* is the set of all triangles, *A*_*i*_ is the area of triangle *i*, *A*_*i*,0_ is the rest area of triangle *i* given by the initial triangulation, *V* is the volume of the cell, *V*_0_ is the preferred volume, and the coefficients *k*_A_ and *k*_V_ control the strength of these constraints. Note that the area constraint acts locally on each triangle, while the volume constraint is applied to the RBC as a whole.

A typical RBC shape is biconcave (see Fig 1), which can be imposed by a combination of the cell area *A*_0_ and volume *V*_0_, and described by a reduced volume as $V_{0}/\left(4\pi R_{0}^{3}/3\right)=0.64$, with $R_{0}=\sqrt{A_{0}/\left(4\pi \right)}$ being the effective RBC radius. The average area of a RBC is equal to about 133 *μm* [45], corresponding to *R*_0_ = 3.25 *μm*. Another important property of a RBC membrane is its stress-free state (or shape), which is referred to the membrane shape with a minimum shear-elasticity energy (zero in our case) and characterized by the choice of the rest lengths *r*_*ij*,0_ of springs. For example, a RBC model in Ref. [31] assumes the rest lengths to be set to the edge lengths of initial triangulation of the membrane biconcave shape, resulting in a biconcave stress-free state. However, recent simulation studies [46, 47] indicate that the stress-free state of a RBC is likely to be a spheroid close to a sphere rather than the biconcave shape and the choice of stress-free state may affect RBC shape and its behavior in flow. In our study, we consider three models (M1, M2, and M3) with two different stress-free states and spontaneous curvatures of a RBC membrane, see Table 1. Stress-free state is characterized by $\nu =V_{s}/\left(4\pi R_{0}^{3}/3\right)$, where *V*_*s*_ is the membrane volume at the stress-free state, while membrane’s spontaneous curvature is dimensionalized as *C*_0_ *R*_0_.

**Table 1:** Different RBC models.

model	stress-free state (*ν*)	spontaneous curvature (*C*_0_ *R*_0_)
M1	0.64	0.0
M2	0.96	0.0
M3	0.96	3.0

Three RBC models with different stress-free states (*ν*) and spontaneous curvatures (*C*_0_ *R*_0_) of a RBC membrane. The model notations (M1, M2 and M3) are used further in text.

### Simulation setup

In simulations, we employ a cubic system with dimensions *L*_*x*_ = *L*_*y*_ = *L*_*z*_ = 14*R*_0_. Periodic boundary conditions (BCs) are assumed in all directions. Note that periodic BCs will affect translational diffusion of a RBC due to finite-size effects, such that the translational diffusion in simulations is slower than that in an infinite system. However, our simulation domain is large enough to have no significant effect on the rotational diffusion of a RBC.

The local area and volume constraint parameters of the cells are set to $k_{A}R_{0}^{4}/\left(k_{B}T\right)=4.2\times 10^{6}$ and $k_{V}R_{0}^{6}/\left(k_{B}T\right)=3.4\times 10^{4}$, respectively. The remaining membrane parameters, bending rigidity *κ* and Young’s modulus *Y*, are expressed through dimensionless numbers as

$$\begin{array}{c} \kappa^{*}=\frac{\kappa }{k_{B}T},\;Y^{*}=\frac{YR_{0}^{2}}{k_{B}T}. \end{array}$$ (17)

Different values for κ* and Y* can be selected. However, κ* ≈ 70 (*κ* ≈ 3 × 10^−19^ J) and Y* ≈ 43680 (*Y* ≈ 18 × 10^−6^ N/m) correspond to average properties of a healthy RBC at a physiological temperature of *T* = 37^*o*^ C [31]. The Young’s modulus can also be related to the membrane shear modulus *μ*. For example, *Y* ≈ 4*μ* for a nearly incompressible membrane [31].

**Fig 2:**
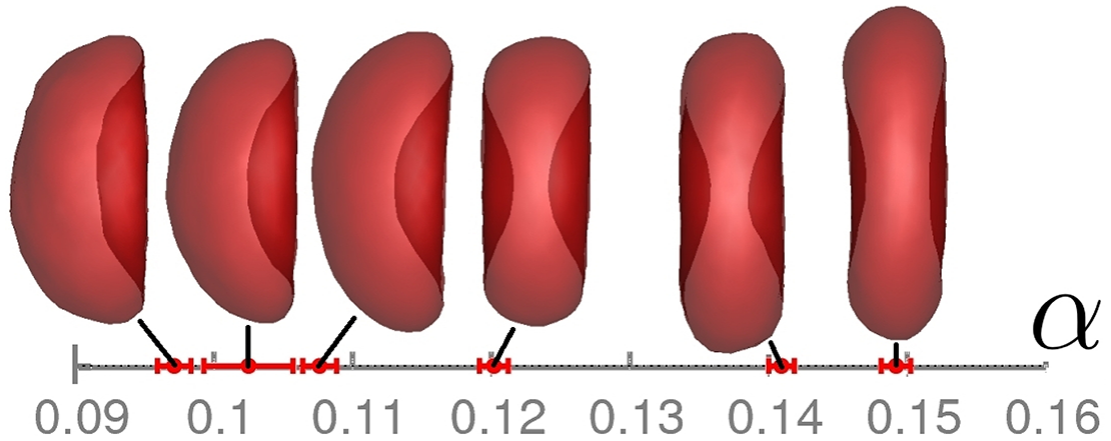
Different RBC shapes. RBC shapes and their asphericities *α* which characterize the deviation from a spherical shape. The asphericity is defined as $\alpha =\left[{\left(\lambda_{1}-\lambda_{2}\right)}^{2}+{\left(\lambda_{2}-\lambda_{3}\right)}^{2}+{\left(\lambda_{3}-\lambda_{1}\right)}^{2}\right]/\left(2R_{g}^{4}\right)$, where *λ*_1_ ≤ *λ*_2_ ≤ *λ*_3_ are the eigenvalues of the gyration tensor of a RBC and $R_{g}^{2}=\lambda_{1}+\lambda_{2}+\lambda_{3}$. The corresponding RBC parameters in the order of ascending asphericity (from a stomatocytic shape to a biconcave shape) are (1) M3, κ* = 20, Y* = 43680; (2) M3, κ* = 40, Y* = 43680; (3) M2, κ* = 70, Y* = 43680; (4) M3, κ* = 70, Y* = 43680; (5) M2, κ* = 20, Y* = 8000; (6) M2, κ* = 70, Y* = 8000. The asphericity values are time-averaged over the whole simulation and the standard deviations are shown as error bars.

Fig 2 presents several RBC shapes for the different models and values of κ* and Y*. The shapes are characterized by asphericity *α*, which describes the deviation from a spherical shape, and range from a stomatocyte (or a cup-like shape) to a biconcave (or discocyte) shape. These combinations of RBC models and membrane properties will be employed further in order to identify potential differences in their SLS and DLS functions. Note that the shape of M1 model always remains biconcave independently of the choice of κ* and Y*, because it coincides with its stress-free state.

For the MPC fluid, we use the average number density of *n* = 20/*a*^3^ solvent particles per collision box, the rotation angle *β* = 130°, and the MPC time step $\Delta t=0.0125\sqrt{m_{s}a^{2}/k_{B}T}$ (*m*_*s*_ = 1, *a* = 1, and *k*_*B*_*T* = 1 are the basic units here). This combination of *n*, *β*, and Δ*t* provides a large enough viscosity of $\eta =138.8\sqrt{m_{s}k_{B}T}/a^{2}$ in order to have a low Reynolds number. In MPC simulations, the effective RBC radius is equal to *R*_0_ = 5.85*a* and the mass of the membrane particles is *m*_m_ = 20*m*_*s*_. The RBC membrane and MPC fluid are coupled dynamically by including the membrane particles into the collision with solvent particles [30, 34, 48], leading to translational and rotational diffusion of the RBC. Solvent particles can cross the membrane; however, no-slip BCs are still accounted for on average due to the membrane-solvent coupling [48]. Also, fluids inside and outside the cell assume the same viscosity.

For the DPD fluid, a number density of *n* = 3/*a*^3^ is employed. The mass of the membrane particles is *m*_m_ = 2*m*_*s*_. Other parameters include the conservative force coefficient *a*_*c*_ = 40*k*_*B*_*T*/*a*, the dissipative coefficient $\gamma =10.0\sqrt{m_{s}k_{B}T}/a$, cutoff radius *r*_*c*_ = 1.5*a* = 0.46*R*_0_, and the exponent *k* = 0.15 for the weight function. These settings yield a fluid viscosity of $\eta =32.85\sqrt{m_{s}k_{B}T}/a^{2}$. A time step of 0.005 is employed, and the simulations are run for at least 6 × 10^7^ time steps to obtain long enough diffusive trajectories. Interactions between the RBC membrane and solvent are mediated by the dissipative force in DPD [31]. In DPD, solvent particles are also able to cross the membrane.

## Results

### Static scattering function

**Fig 3:**
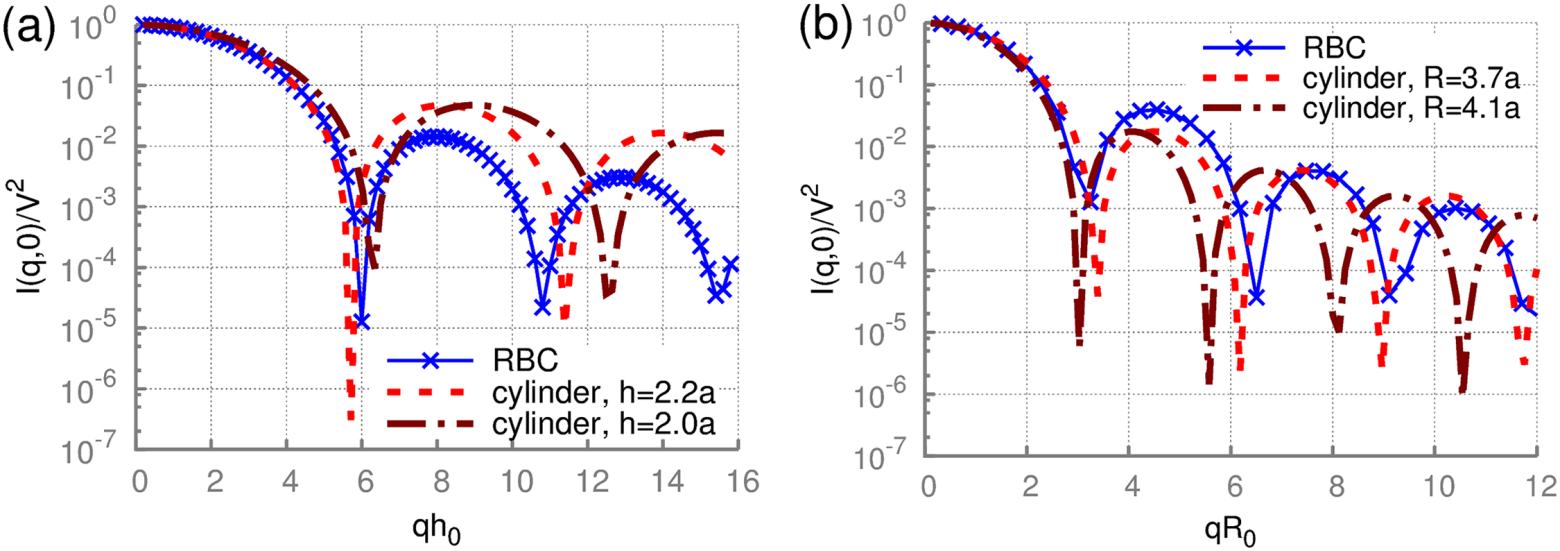
Static scattering by a RBC with fixed orientation. The scattering intensity *I* = AA* of a rigid discocyte for wave vectors **q** (a) parallel and (b) perpendicular to the RBC axis of rotational symmetry. *h*_0_ = 2*a*. Analytical solutions for a cylinder (see S3 Appendix) with different radii *R* and heights *h* are also plotted for comparison.

The static scattering function for a RBC can be readily computed using a triangulated discocyte shape [49], as shown in Fig 1. The numerical evaluation of the scattering amplitude *A*(**q**, *t*) for a triangulated surface is described in S1 Appendix, while the correctness of our implementation is verified for a cylindrical shape in S3 Appendix. Fig 3 presents the scattering intensity *I* = AA* for the biconcave RBC shape (i.e., rigid cell) and fixed directions of the wave vector **q** relative to the cell’s symmetry axis. The RBC intensity is compared to the analytical solutions for a cylinder (see S3 Appendix) with radii *R* and heights *h*, chosen such that the first minima approximately coincide. Clearly, there are some similarities as the discocyte shape of a RBC geometrically resembles a short cylinder. Pronounced differences between the scattering intensities of a RBC and a cylinder appear at higher *q* values, which are more sensitive to small features of the biconcave RBC shape. However, the first and second minima for RBC-shape intensity can be matched with the theory to produce estimates of the height *h* and radius *R* of the discocyte. These values match the dimensions of the discocyte very closely.

Fig 3 corresponds to scattering from a rigid RBC with a fixed orientation in space (e.g., adhered to a surface). However, our main interest is in the scattering properties of soft RBCs in dilute solution, where arbitrary orientations of the cells are equally probable due to rotational diffusion and RBCs are subject to membrane fluctuations; this setting also corresponds to typical experimental situations. In simulations, different cell orientations correspond to a number of randomly-selected initial conditions for the RBC orientation, which we realize not by making multiple simulations for the different initial conditions, but by performing the orientational average over a number of random **q**-vector orientations *N*_avg_, as given in Eq (6) and S2 Appendix. Furthermore, to take into account RBC membrane fluctuations, we perform equilibrium simulations of a deformable RBC diffusing in a solvent; these simulations are used for the evaluation of both SLS and DLS functions.

**Fig 4:**
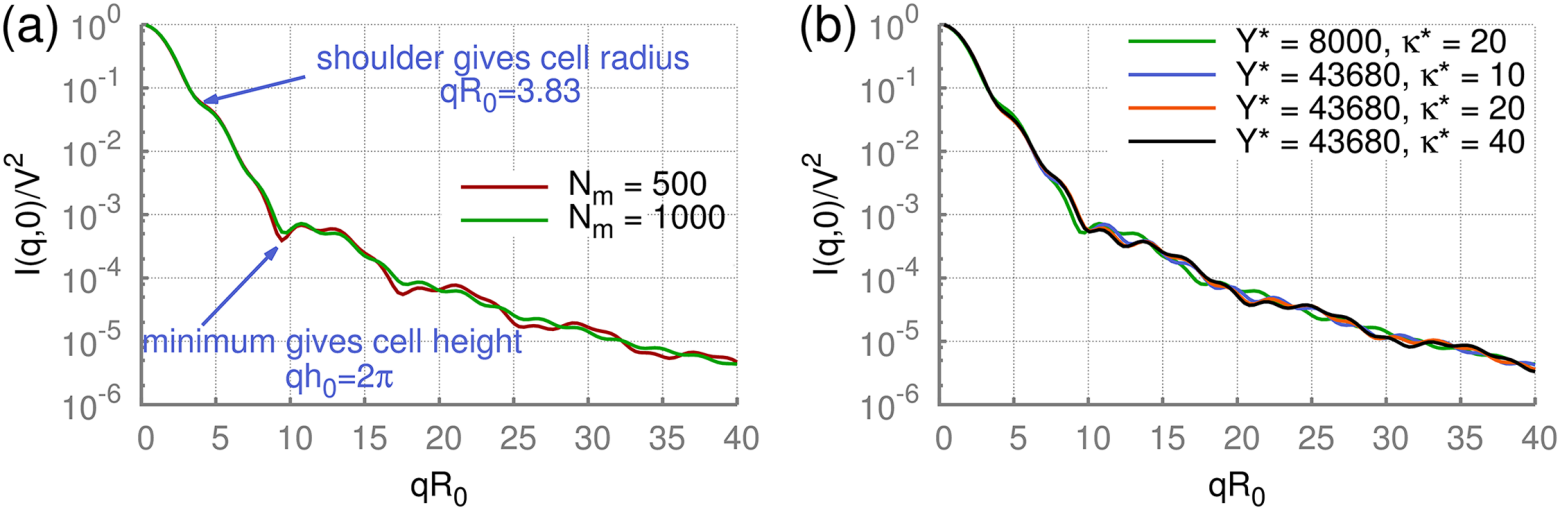
Orientationally-averaged static scattering by a RBC. Orientationally-averaged static scattering functions of RBCs for different parameters using the DPD method with RBC model M3. (a) Effect of surface discretization (*N*_m_ = 500 and *N*_m_ = 1000) on static scattering intensity. Model M3 with κ* = 20 and Y* = 8000. (b) Effect of bending rigidity and shear modulus on the static scattering intensity. Cells with Y* = 8000 remain biconcave, whereas those with the large value of Y* = 43680 attain a stomatocytic shape in simulations.

The computed static scattering intensity for a soft RBC is shown in Fig 4(a) for two different discretizations characterized by the number of vertices *N*_m_. The orientationally-averaged scattering intensity is obviously different from the case of a fixed orientation; however, a careful look at these curves still allows the recognition of characteristic features. First, a shoulder in the scattering intensity at *qR*_0_ ≈ 3.83 is clearly visible in all cases, which corresponds to the RBC radius. Second, the scattering curves have a local minimum at *qh*_0_ = 2*π* related to the thickness of RBCs. Thus, the two main geometrical characteristics of RBCs are still visible in the orientationally averaged scattering intensities. Furthermore, the level of discretization can also have an effect mainly at high *q* values. Different surface discretizations may lead to slightly different RBC shapes and to very small differences in cell volume. However, the scattering curves for discretizations with a number of points larger than *N*_m_ = 1000 become nearly independent of *N*_m_. Therefore, we generally employ *N*_m_ = 1000 further in simulations.

Fig 4(b) presents several static-scattering curves for different membrane bending rigidities and Young’s moduli. The scattering intensities for the large Young’s modulus, Y* = 43680, and various κ* values do not show significant differences, indicating that the overall RBC shape determines static measurements and thermal undulations of the membrane have a very weak effect. The comparison of scattering intensities for different Young’s moduli shows stronger differences than for various bending rigidities. Note that for the larger Y* value, RBCs do not remain biconcave, but attain a weak stomatocytic (or a cup-like) shape, while simulations with the smaller Y* lead to a biconcave cell shape, see Fig 2. As the result, the differences in scattering intensities for different Young’s moduli in Fig 4(b) are mainly due to a change in shape. However, differences in the static scattering function for biconcave and stomatocytic RBC shapes are not very pronounced, and therefore the orientationally-averaged SLS function is expected to be not very sensitive to the changes in membrane properties and moderate alterations in cell shapes.

### Intermediate scattering function

**Fig 5:**
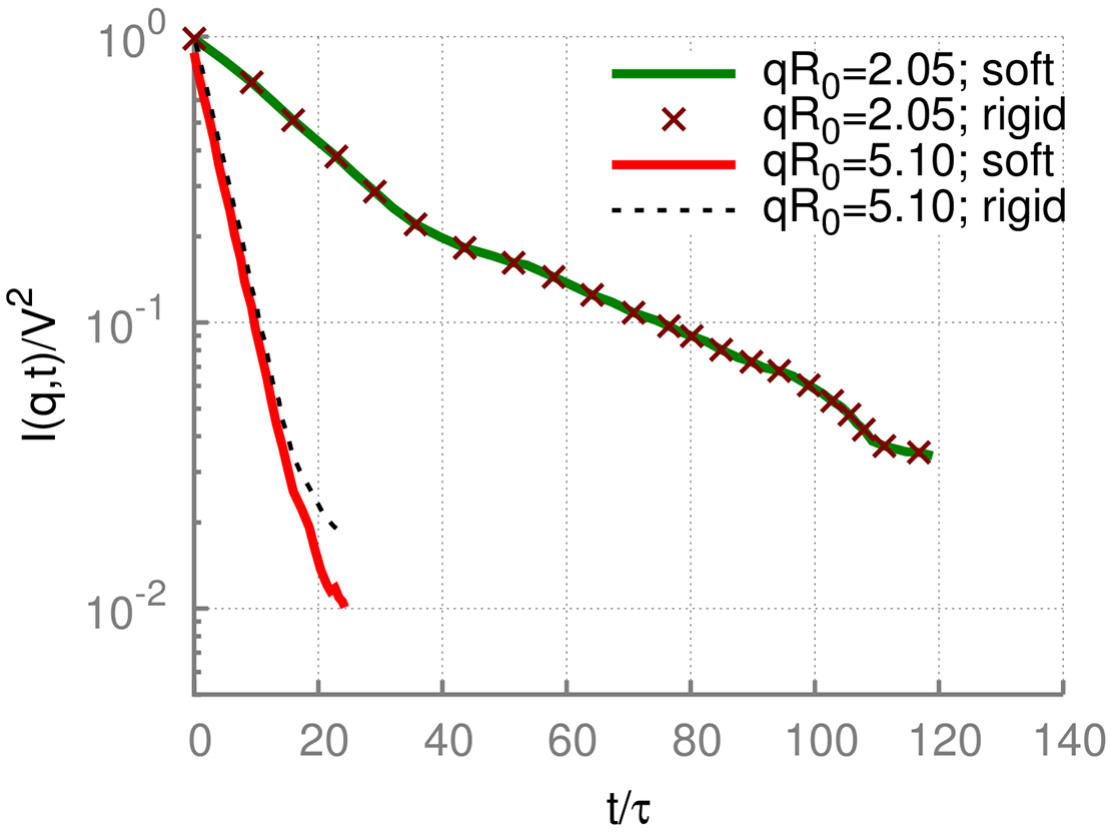
Intermediate scattering measurements. Intermediate scattering functions for two selected *q* values calculated through the orientational averaging. The curves for deformable RBCs were obtained directly from MPC simulations of a diffusing RBC (model M1 with *N*_m_ = 500, κ* = 20, and Y* = 8000). The curve for a rigid cell was obtained by the substitution of all cell snapshots in time within the trajectory for a deformable RBC with a rigid biconcave shape through matching instantaneous cell’s center of mass and orientation. Time is normalized by a characteristic relaxation time *τ* = *ηR*_0_/*Y* of a RBC. For typical values of RBC elasticity (*Y* = 18.9 × 10^−6^ *N*/*m* [31]) and plasma viscosity (*η* = 1.2 × 10^−3^ *Pa*⋅*s* [45]), *τ* ≈ 2.1 × 10^−4^ *s*.

In order to access dynamical properties of diffusing RBCs, we evaluate the intermediate scattering function (Eq (2)) from simulations of RBC diffusion, followed by an orientational averaging, as described in Sec. Theoretical Background and S2 Appendix. The intermediate scattering function *I*(*q*, *t*) obtained from MPC simulations is plotted in Fig 5 for two different wave numbers *q*. For the both values of *q*, an initial exponential decay is observed, which readily yields *D*_eff_(*q*). At larger times *t*, there is typically a crossover to a slower decay, which we do not take into consideration. All other values of *q* (not shown here) display a similar behavior.

Fig 5 also compares the intermediate scattering functions for deformable and rigid cells. The curves for deformable RBCs have been computed directly from simulations of a diffusing RBC. In the case of rigid cells, we have used the trajectory of positions and orientations for deformable cells, but substituted at all instances in time the actual cell shape by a fixed rigid biconcave shape with matching center of mass and orientation. For *qR*_0_ = 2.05 in Fig 5, the curves for deformable and rigid cells completely overlap within numerical accuracy. For *qR*_0_ = 5.1, some differences at long correlation times between deformable and rigid cells are visible; however, the initial exponential decay remains same independently of the cell’s stiffness. The good agreement between the intermediate scattering functions for deformable and rigid cells at low *q* indicates that the DLS function is insensitive to thermal fluctuations of a membrane in this q-range.

**Fig 6:**
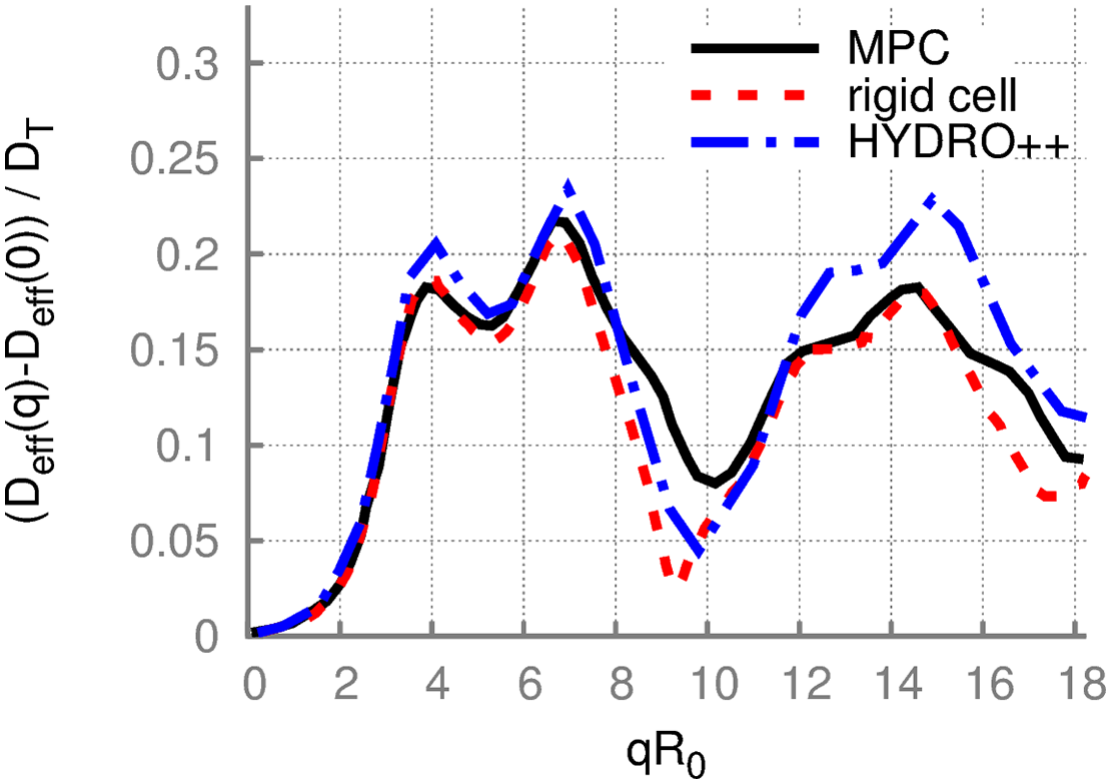
Effective diffusion of a RBC from MPC simulations. The dimensionless effective diffusion coefficient $D_{\text{eff}}^{*}\left(q\right)$ of a RBC as a function of *q*, obtained from different methods. MPC simulation results correspond to a deformable cell represented by model M1 with *N*_m_ = 500, κ* = 20, and Y* = 8000 (black line) and to a superimposed rigid cell using the simulated trajectory of the deformable RBC (red line). The curve from HYDRO++ is for a rigid cell (blue line). The data are averaged over *N*_avg_ = 2000 random q-vector orientations, see S2 Appendix.

From *I*(*q*, *t*), we can determine the exponential decays at short times, and therefore the effective diffusion coefficient given by Eq (4). Fig 6 presents the dimensionless effective diffusion coefficient

$$\begin{array}{c} D_{\text{eff}}^{*}\left(q\right)\equiv \frac{D_{\text{eff}}\left(q\right)-D_{\text{eff}}\left(0\right)}{D_{T}}\;, \end{array}$$ (18)

where *D*_*T*_ is the translational diffusion coefficient of a RBC. This dimensionless presentation of the effective diffusion coefficient facilitates an easy comparison of results obtained from different methods and for various (outside) fluid viscosities.

The effective diffusion coefficient *D*_eff_(*q*) in the limit of *q* → 0 should yield the translational diffusion coefficient of a RBC. Extrapolation of *D*_eff_(*q*) to *D*_eff_(0) provides a value which agrees within a few percent with *D*_*T*_ obtained directly from the mean-squared displacement of a RBC. The dependence of *D*_eff_(*q*)* on *q* in Fig 6 clearly shows that the non-translational contributions to *D*_eff_(*q*)* already appear at *qR*_0_ ≈ 2, while the first maximum is observed at approximately *qR*_0_ ≈ 4. These features have their origin either in the rotational dynamics of a RBC or membrane undulations due to the softness of a membrane. To distinguish these two potential contributions, we employ again the full simulated trajectory for a deformable RBC and replace all snapshots of the soft cell in time with the rigid biconcave shape of the cell following its center-of-mass position and orientation. Thus, we construct a trajectory of the solid RBC with the same translational and rotational diffusion characteristics as the original trajectory for the deformable cell. *D*_eff_(*q*)* for the superimposed rigid RBC in Fig 6 indicates that most features of the *D*_eff_(*q*)* curves for soft and solid RBCs are the same, which implies that rotational diffusion provides the dominant contribution, especially at low *q* values. The effect of membrane undulations mainly appears at high *q* values. The most prominent difference between the *D*_eff_(*q*)* curves for soft and solid RBCs is found at a local minimum at approximately *qR*_0_ ≈ 10.

In order to confirm the results obtained from the MPC simulations, we have also employed another method, where the effective diffusion coefficient is computed directly from the diffusion matrix of the discocyte in the program HYDRO++ [50, 51] (for details of the calculation, see S4 Appendix). This approach uses a different description of the cell shape such that a RBC is modeled by a collection of beads which fill the cell volume. The results from HYDRO++ are also shown in Fig 6 and match those for the solid erythrocyte very well for $qR_{0}≲12$. We attribute the deviations for $qR_{0}≳12$ to the different representation of the cell shape in triangulated membranes and bead packings.

**Fig 7:**
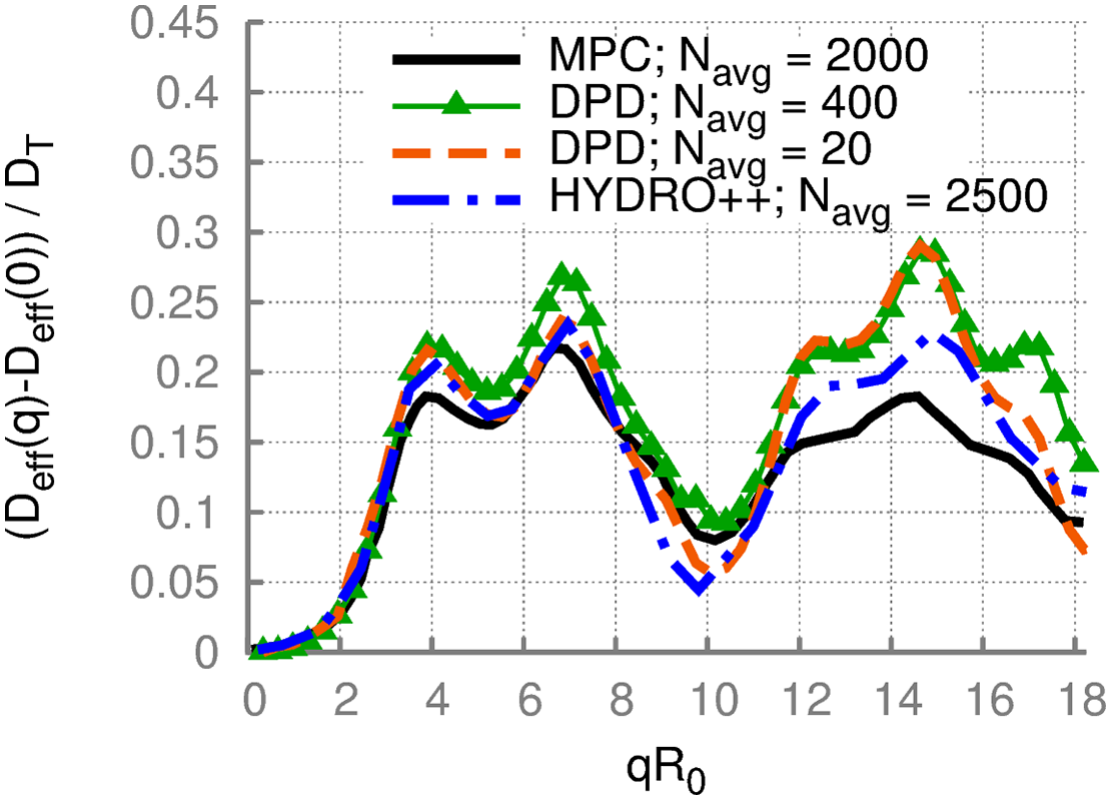
Comparison of DPD and MPC results. The dimensionless effective diffusion coefficient *D*_eff_(*q*)* of a soft RBC represented by model M1 with a bending rigidity κ* = 20. Two different curves for DPD simulations correspond to different numbers *N*_avg_ of random orientations of **q** vector used for averaging. For comparison, MPC results are also shown. In DPD, the Young’s modulus of the RBC membrane is set to Y* = 43680, whereas in MPC Y* = 8000.

Fig 7 presents the dimensionless effective diffusion coefficient for a soft RBC simulated with the DPD method. The DPD results have been obtained with either *N*_avg_ = 20 or *N*_avg_ = 400 random orientations of **q** vector for averaging. This means that for every selected direction of **q** vector time correlations of the scattering amplitude are calculated and then averaged over all orientations. We have verified that about *N*_avg_ = 400 orientations is sufficient to represent an isotropically-averaged converged function for the scattering amplitudes. The comparison of DPD results with MPC in Fig 7 is rather good, even though some deviations, especially at high *q* values, are clearly visible. At high *q* values, small features such as membrane fluctuations become important. Note that the RBC membrane in DPD simulations has a Young’s modulus of Y* = 43680, which is larger than Y* = 8000 used in the MPC simulations. We attribute discrepancies between the MPC and DPD results at high *q* values to this difference in mechanical properties of the membrane. Interestingly, the curve obtained by HYDRO++ fits the DPD results better than MPC results, see Fig 7.

**Fig 8:**
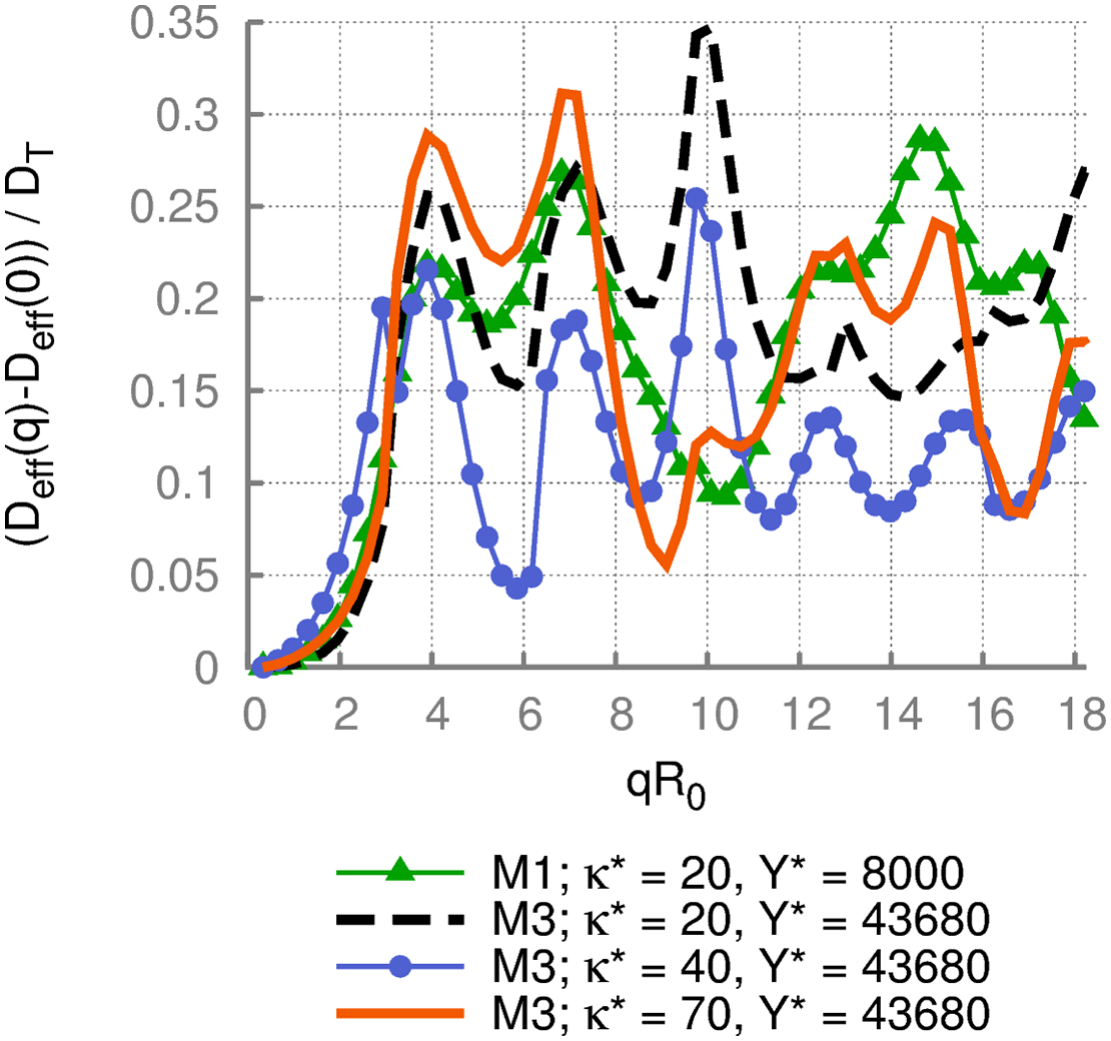
Effective diffusion of a RBC for various membrane properties. The dimensionless effective diffusion coefficient *D*_eff_(*q*)* of RBCs with different membrane properties. The cells differ in bending rigidity κ*, Young’s modulus Y*, and spontaneous curvature. Two cells remain biconcave, whereas the other two attain a cup shape, which is reflected in the third peak at *qR*_0_ ≈ 10.

Fig 8 shows the dimensionless effective diffusion coefficient for cells with different membrane properties. The cells differ in bending rigidity κ*, Young’s modulus Y*, and spontaneous curvature. The data for model M1 with κ* = 20 and Y* = 8000 is the same as ‘DPD, *N*_avg_ = 400’ in Fig 7. It is important to note that two cells (M1 with κ* = 20 and Y* = 8000; M3 with κ* = 70 and Y* = 43680) remain biconcave, while the other two attain a cup shape, see Fig 2. This change in shape from discocyte to cup-like is nicely reflected in *D*_eff_(*q*)* by the appearance of a third peak at *qR*_0_ ≈ 10. Thus, Fig 8 clearly illustrates the effect of the cell shape on dynamic light scattering signals.

## Discussion

Static light scattering is a well-established tool for assessing structural properties of suspended particles. However, our results for diffusing RBCs indicate that orientationally-averaged SLS measurements are insensitive to moderate differences in cell shapes (e.g., biconcave and cup-like). SLS experiments performed on malaria-infected RBCs [10] and on RBCs in sickle-cell anemia [16] have demonstrated that differences in RBC shapes induced by these diseases can be detected. The main difference between these experiments and our study is that in the experiments, RBCs were lying on a coverslip such that scattering measurements for a fixed orientation have been performed, while in our simulations we compute static scattering from a diffusing RBC, so that it is averaged over all possible cell orientations. A disadvantage of fixing cell orientation in comparison to monitoring a freely diffusing cell is that a method for fixing cell’s position (e.g., cell adhesion) may alter cell’s natural shape as well as trigger some undesired dynamic response. However, as our SLS results have shown, SLS measurements for the differentiation of moderate changes in RBC shapes are expected to be useful primarily in the case of a fixed cell orientation. Furthermore, SLS measurements on a diffusing RBC are insensitive to thermal undulations of the membrane, and thus cannot be employed for the quantification of membrane properties.

Dynamic light scattering provides information about the cell dynamics and membrane properties. Clearly, one of the straightforward measurements is the translational diffusion of RBCs, which can be obtained from the effective diffusion coefficient *D*_eff_(*q*) in the limit of *q* → 0. DLS experiments with RBCs lying on a coverslip (i.e., with a fixed cell orientation) [10, 11] demonstrate the possibility to measure thermal undulations of a RBC membrane. Following these measurements, it should be possible to deduce cell’s membrane properties, since RBC membrane fluctuations are directly correlated with membrane mechanical characteristics and potential cell activity [18, 52]. Another example of the quantification of DLS measurements is the determination of rod length in a suspension [7], since for rods a reliable theory exists. Here, we provide a step toward the theoretical background for a quantitative interpretation of DLS measurements for soft particles such as RBCs.

The dimensionless effective diffusion presented in Figs 6–8 shows no significant sensitivity to membrane properties at low *q* values, indicating that this range of wave vectors describes an overall diffusive behavior of a RBC including its translational and rotational contributions. Furthermore, the scattering functions obtained from different simulation methods are found to be nearly independent of the method employed. An interesting observation in Fig 8 is the appearance of an additional peak at *qR*_0_ ≈ 10 for cells with a stomatocytic shape, which allows the differentiation between stomatocytic and biconcave RBC shapes. Furthermore, at large enough *q* values, significant differences for various conditions were detected. These differences in *D*_eff_(*q*)* at large *q* are attributed to differences in membrane properties and can potentially be employed to infer cell’s characteristics from DLS experimental measurements. Thus, our simulation results show that quantitative interpretation of DLS measurements is possible, and DLS experiments are needed to ascertain the accuracy and applicability of the simulation measurements.

Although the presented simulation results are promising, it is worthwhile to discuss some limitations of the current simulations. We have employed the Rayleigh-Gans-Debye approximation [1] for the calculation of scattering, which is based on the assumption of elastic scattering preserving the wave length. It is not clear whether this approximation is appropriate and good enough, a question which can be clarified by corresponding experimental measurements. There also exist other methods for the calculation of scattering such as, for instance, discrete dipole [53] and Born [54] approximations. The discrete dipole approximation permits the setting of the refraction index individually for different parts of the scattering object. Other simplification we have made is the assumption of no light absorption and no refraction on a cell membrane. Finally, we have neglected any multiple scattering effects, which is a reasonable assumption for a dilute solution of RBCs. Some of these simplifications may not be critical for scattering results, while other mentioned limitations can be elevated by improvements of the modeling approach.

In conclusion, realistic modeling of scattering intensities has a great potential for the understanding and quantification of scattering signals obtained experimentally. In particular, for blood a light scattering analysis offers prospects of non-invasive *in vitro* and *in vivo* means to detect and identify pathological states of blood cells and/or the presence of disease-related objects (e.g., bacteria, virus) in blood. Here, an interesting direction is to investigate DLS of dense suspension of RBCs under flow, which is already possible from the modeling standpoint [55, 56]. Clearly, this direction requires the development, testing, and validation of new and existing simulation and experimental models and approaches. We hope that our results will motivate further simulation studies of light scattering, since simple analytical models are likely to suffer from serious shortcomings when used for the quantitative interpretation of scattering by complex biological systems.

## Supporting information

S1 Appendix Scattering amplitude. (PDF)

S2 Appendix Orientational average. (PDF)

S3 Appendix Static scattering from a cylinder. (PDF)

S4 Appendix Bead model calculation of effective diffusion. (PDF)
