# Supplementary material for: Static and dynamic light scattering by red blood cells: A numerical study
Source: PLoS One. 2017 May 4;12(5):e0176799. doi: 10.1371/journal.pone.0176799 (PMC5417630; doi:10.1371/journal.pone.0176799)
Supplement: S1 Appendix — (PDF) [file pone.0176799.s001.pdf]

S1 Appendix

Scattering amplitude

Given a triangulated RBC surface, we employ the following procedure [47] to evaluate numerically the scattering amplitude from Eq. (1) in the main text. First, the volume integral can be expressed in terms of a surface integral using the divergence theorem as

$$A(\mathbf{q},t) = -i\int_S \frac{\mathbf{n}\cdot\mathbf{q}}{q^2}e^{i\mathbf{q}\cdot\mathbf{r}}\mathrm{d}^2r, \tag{S1-1}$$

where the integral is now over the closed surface S of the RBC membrane. This integral can be immediately simplified further by expressing it as a sum over triangles as

$$A(\mathbf{q},t) = -i\sum_j \frac{\mathbf{n}_j\cdot\mathbf{q}}{q^2}\int_{S_j} e^{i\mathbf{q}\cdot\mathbf{r}}\mathrm{d}^2r, \tag{S1-2}$$

where S_j and $\mathbf{n}_j$ are the surface and outward-facing normal of triangle j, respectively.

The above integral over a triangular surface can easily be computed [47]. Let us consider the scattering intensity from a wave vector $\mathbf{q}$ at a triangle S_{tri} whose corners are given by the position vectors $\mathbf{r}_1$, $\mathbf{r}_2$, and $\mathbf{r}_3$. The scattering amplitude from this triangle is given by the integral

$$A_{\mathrm{tri}}(q,\mathbf{r}_1,\mathbf{r}_2,\mathbf{r}_3) = \int_{S_{\mathrm{tri}}} e^{i\mathbf{q}\cdot\mathbf{r}}\mathrm{d}^2r. \tag{S1-3}$$

In order to evaluate the integral in Eq. (S1-3), we parametrize the surface of the triangle as

$$\mathbf{r} = \mathbf{r}_1 + \xi_1(\mathbf{r}_2-\mathbf{r}_1) + \xi_2(\mathbf{r}_3-\mathbf{r}_1) \tag{S1-4}$$

so that Eq. (S1-3) can be written as an integral over ξ_1 and ξ_2 as

$$A_{\mathrm{tri}} = \int_0^1 \mathrm{d}\xi_1 \int_0^{1-\xi_1} \mathrm{d}\xi_2 \sqrt{\det\rho}\, e^{i\mathbf{q}\cdot[\mathbf{r}_1+\xi_1(\mathbf{r}_2-\mathbf{r}_1)+\xi_2(\mathbf{r}_3-\mathbf{r}_1)]}, \tag{S1-5}$$

where the determinant of the metric tensor ρ is given by

$$\sqrt{\det\rho} = \left|(\mathbf{r}_2-\mathbf{r}_1)\times(\mathbf{r}_3-\mathbf{r}_1)\right|. \tag{S1-6}$$

Equation (S1-5) can be evaluated as

$$\begin{aligned} A_{\mathrm{tri}}(q,\mathbf{r}_1,\mathbf{r}_2,\mathbf{r}_3) = \sqrt{\det\rho}\,\Bigg[&\frac{\exp(i\mathbf{q}\cdot\mathbf{r}_1)}{\mathbf{q}\cdot(\mathbf{r}_1-\mathbf{r}_3)\mathbf{q}\cdot(\mathbf{r}_2-\mathbf{r}_1)} + \\ &\frac{\exp(i\mathbf{q}\cdot\mathbf{r}_2)}{\mathbf{q}\cdot(\mathbf{r}_2-\mathbf{r}_1)\mathbf{q}\cdot(\mathbf{r}_3-\mathbf{r}_2)} + \frac{\exp(i\mathbf{q}\cdot\mathbf{r}_3)}{\mathbf{q}\cdot(\mathbf{r}_3-\mathbf{r}_1)\mathbf{q}\cdot(\mathbf{r}_2-\mathbf{r}_3)}\Bigg]. \end{aligned} \tag{S1-7}$$
