# Supplementary material for: Static and dynamic light scattering by red blood cells: A numerical study
Source: PLoS One. 2017 May 4;12(5):e0176799. doi: 10.1371/journal.pone.0176799 (PMC5417630; doi:10.1371/journal.pone.0176799)
Supplement: S2 Appendix — (PDF) [file pone.0176799.s002.pdf]

S2 Appendix

Orientational average

An orientational average of the static and intermediate scattering functions has to be performed. This means that the integral in Eq. (6) of the main text is evaluated over many spatial directions in order to make the scattering amplitude independent of **q**-vector orientation and dependent only on a scalar value of q. For this purpose, we define a set of N_{avg} points chosen randomly and uniformly at a spherical surface with the corresponding directions $\{(\theta_i, \phi_i)\}_{i=1}^{N_{\text{avg}}}$ in spherical coordinates. For a large enough number N_{avg}, the orientational average is approximated as

$$\int_{\Omega} I(\mathbf{q}, t) \mathrm{d}\Omega \approx \frac{1}{N_{\text{avg}}} \sum_{i} I(\mathbf{q}_i, t), \qquad \text{(S2-1)}$$

where $\mathbf{q}_i$ is a vector with a direction (θ_i, ϕ_i) in spherical coordinates and the absolute value $q = |\mathbf{q}_i|$. For simulations with a duration comparable to one rotational diffusion time of a RBC, we have confirmed that $N_{\text{avg}} \approx 400$ is large enough to properly capture the orientational average, which becomes independent of the point set selected.
