# Supplementary material for: Static and dynamic light scattering by red blood cells: A numerical study
Source: PLoS One. 2017 May 4;12(5):e0176799. doi: 10.1371/journal.pone.0176799 (PMC5417630; doi:10.1371/journal.pone.0176799)
Supplement: S3 Appendix — (PDF) [file pone.0176799.s003.pdf]

S3 Appendix

Static scattering from a cylinder

To verify the numerical evaluation of the scattering amplitude $A(\mathbf{q}, t)$ described in S1_Appendix, we consider a cylinder shape, because it yields an analytical solution for certain cylinder orientations or directions of the $\mathbf{q}$ vector. We define a cylinder with radius R and axis in the z-direction. The height of the cylinder is equal to h and it is placed such that $-h/2 < z < h/2$. When the vector $\mathbf{q}$ is parallel to the cylindrical axis (i.e., $\mathbf{q} = q\mathbf{e}_z$), the scattering amplitude is obtained to be

$$A^{\parallel}(q) = 2\pi R^2 \frac{\sin(qh/2)}{q}. \tag{S3-1}$$

This immediately shows that the scattering amplitude, and thus the scattering intensity $I = AA^*$ both have the first minimum at $q = 2\pi/h$. When the vector $\mathbf{q}$ is perpendicular to the cylinder axis (e.g., $\mathbf{q} = q\mathbf{e}_x$), the scattering amplitude becomes

$$A^{\perp}(q) = \frac{2\pi h R J_1(qR)}{q}, \tag{S3-2}$$

where J_1 is the Bessel function of first kind. Thus, in this case, the scattering amplitude and intensity reach their first minimum at the first zero of J_1 at $qR \approx 3.83$.

The calculation of scattering amplitude given in S1_Appendix requires a triangulated surface. To triangulate the surface of a cylinder, we first discretize the cylinder side with a number of rectangles whose one side is equal to h. Then, each rectangle is divided into two triangles separated by a diagonal. The top and bottom of the cylinder are triangulated by connecting the vertices of rectangles with the top and bottom centers. Figure S3-1 shows the comparison of scattering intensities of a cylinder obtained from Eqs. (S3-1) and (S3-2) and by numerical integration over a triangulated cylinder surface with $R = 5a$ and $h = 20a$. Numerical results are in excellent agreement with the corresponding analytical solutions, verifying the correctness of our implementation for the calculation of scattering amplitudes.

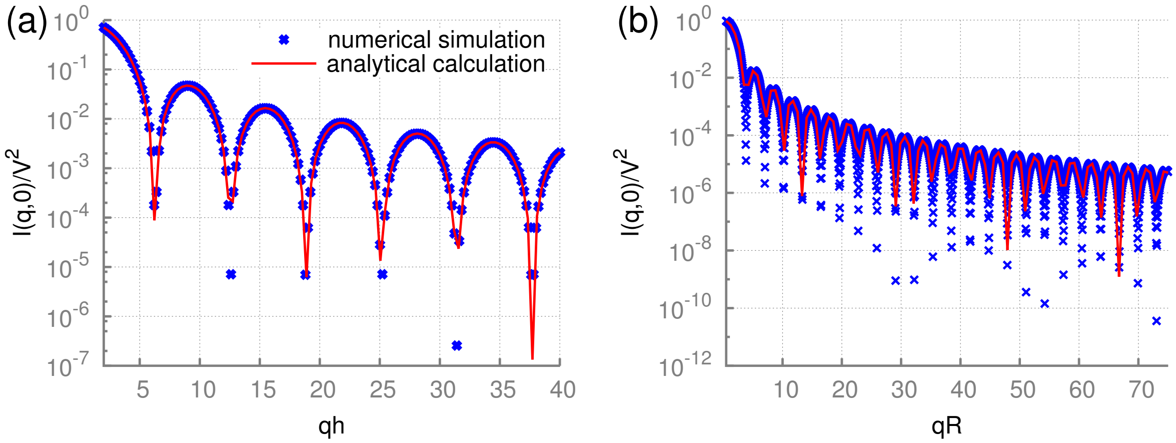

Fig. S3-1. Static scattering from a fixed cylinder. The scattering intensity $I = AA^*$ of a cylinder with a radius $R = 5a$ and height $h = 20a$ for wave vectors $\mathbf{q}$ (a) parallel and (b) perpendicular to the axis of the cylinder. The intensity is calculated analytically using Eqs. (S3-1) and (S3-2) as well as numerically following the approach described in S1_Appendix.
