# Supplementary material for: Static and dynamic light scattering by red blood cells: A numerical study
Source: PLoS One. 2017 May 4;12(5):e0176799. doi: 10.1371/journal.pone.0176799 (PMC5417630; doi:10.1371/journal.pone.0176799)
Supplement: S4 Appendix — (PDF) [file pone.0176799.s004.pdf]

S4 Appendix

Bead model calculation of effective diffusion

The effective diffusion coefficient D_{eff} of a rigid structure defined by a set of beads or spheres with positions $\mathbf{r}_i$ can be obtained from the following expression

$$D_{\text{eff}}(q) = \frac{1}{q^2 S(q)} \sum_{j,k} \left\langle b_j b_k e^{-i\mathbf{q}\cdot\mathbf{r}_j} \begin{pmatrix} \mathbf{q} \\ \mathbf{q}\times\mathbf{r}_j \end{pmatrix} \mathbf{D} \begin{pmatrix} \mathbf{q} \\ \mathbf{q}\times\mathbf{r}_k \end{pmatrix} e^{i\mathbf{q}\cdot\mathbf{r}_k} \right\rangle \tag{S4-1}$$

where $\mathbf{D}$ is the diffusion matrix, $S(q)$ is the static form factor, and b_j is the scattering length of particle j. A bead model of the discocyte can be constructed by taking a volume occupied by a hcp-lattice of particles with a radius r_b and removing all particles outside the discocyte volume. The diffusion matrix has been calculated based on the discocyte-shaped set of particles using the software HYDRO++ [48,49]. In this program, hydrodynamic interactions between the beads from the set are introduced using the Kirkwood-Riseman approximation with a volume correction for the calculation of rotational properties [48]. The hydrodynamic radius of the particles takes the values $5a$, $3a$, $2a$, and $1.5a$. D_{eff} converges nicely below $r = 2a$, and it changes by less than 0.5% when $r \approx 1.5a$, which corresponds to the data set in Fig. 6 of the main text. There is a good agreement between both translational and rotational diffusion coefficients in comparison with MPC and DPD simulations. The orientational average was taken over 2500 $\mathbf{q}$-vectors.
